# On the ecological duality between ciliates and dinoflagellates across marine ecosystems

**DOI:** 10.1093/plankt/fbaf049

**Published:** 2025-09-21

**Authors:** Albert Calbet

**Affiliations:** Department of Marine Biology and Oceanography, Institut de Ciències del Mar, Consejo Superior de Investigaciones Científicas, Passeig Marítim de la Barceloneta 37-49, Barcelona 08003, Spain

**Keywords:** microzooplankton, ciliates, dinoflagellates, seasonal succession, mixotrophy, ocean stratification, nutrient dynamics, climate change

## Abstract

Marine ciliates and dinoflagellates are key microzooplankton groups in oceanic food webs. A prevailing ecological framework suggests that ciliates dominate under cool, mixed conditions typical of late winter and early spring, whereas dinoflagellates prevail in warmer, stratified waters during late spring and summer. This review highlights how temperature, stratification, nutrient dynamics, prey composition, turbulence and top–down control shape seasonal and regional patterns. While the ciliate–dinoflagellate succession is often observed in temperate seas, it is not universal. Polar regions exhibit compressed seasonality, while tropical systems show weak seasonality, often dominated by mixotrophic dinoflagellates. The widespread occurrence of mixotrophy in both groups complicates this duality, allowing species to persist across contrasting environmental conditions. Ultimately, the relative dominance of ciliates or dinoflagellates reflects a context-dependent interplay of multiple drivers rather than a fixed seasonal rule. As climate change intensifies ocean stratification and alters nutrient regimes, understanding these dynamics becomes critical to predict shifts in plankton communities and their consequences for marine biogeochemical processes and ecosystem functioning.

## INTRODUCTION

**Fig. 1:**
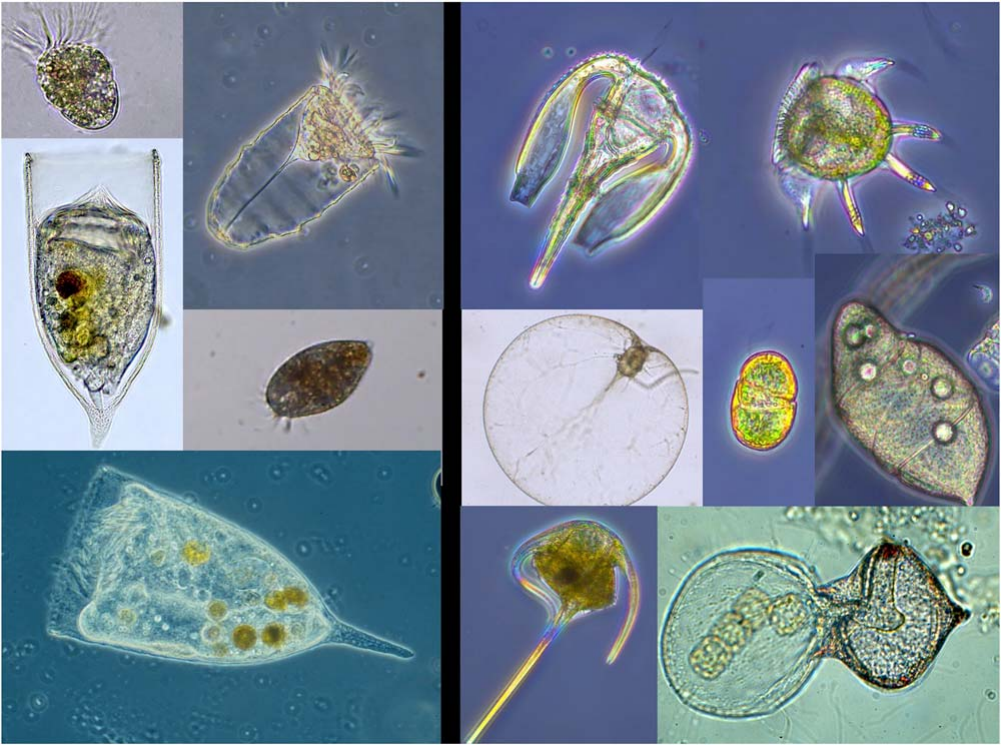
Ciliates (left) and dinoflagellates (right).

Microzooplankton are generally defined (*sensu lato*) as heterotrophic and mixotrophic protists between approximately 20 and 200 μm in size (although they may include a wider array of larger and smaller forms). They primarily comprise ciliates and heterotrophic dinoflagellates (Fig. 1) together with a diverse cast of other protists such as flagellates, acantharians, radiolarians and foraminiferans. These organisms are remarkably abundant in marine waters and, despite their tiny individual biomass, their collective influence is substantial. It is estimated that microzooplankton consume roughly 60–70% of daily oceanic primary production, exerting intense grazing pressure on phytoplankton communities (Calbet and Landry, 2004; Schmoker *et al.*, 2013). Their feeding regulates primary-producer populations, maintains microbial-web balance and shapes the efficiency of carbon transfer to higher trophic levels (Sherr and Sherr, 2002; Calbet and Saiz, 2005; Saiz and Calbet, 2007).

Among the microzooplankton, two prominent groups—ciliates and dinoflagellates—play key ecological roles that often alternate or overlap throughout seasonal cycles. Ciliates are protists characterized by cilia used for locomotion and feeding, usually on small prey such as bacteria and flagellates. They exhibit diverse feeding strategies and occupy a variety of niches, from purely heterotrophic to mixotrophic lifestyles. Mixotrophic ciliates (e.g. *Mesodinium rubrum*, *Laboea*, *Strombidium*) practice kleptoplasty: they retain cryptophyte chloroplasts—and sometimes nuclei—for days to weeks, so continued phagotrophy is required to renew their photosynthetic toolkit (Johnson *et al.*, 2007; Johnson, 2011). Ciliates are known for rapid growth rates and can respond swiftly to phytoplankton blooms, effectively regulating algal abundance under bloom conditions (Montagnes *et al.*, 2010; Vaqué *et al.*, 1997).

Dinoflagellates, on the other hand, are notable for their flexibility in prey selection, consuming a broad spectrum of prey sizes and types, from small algae to larger diatoms and even ciliates themselves. They span the full trophic continuum: photoautotrophs, constitutive mixotrophs that house permanent plastids, non-constitutive mixotrophs that sequester transient plastids or prey cytoplasm, and obligate heterotrophs (Taylor, 1987; Stoecker, 1999; Jeong *et al.*, 2010). Small heterotrophic or plastid-retaining dinoflagellates (<30 μm) directly overlap ciliates in prey size, whereas larger raptorial forms deploy peduncle or pallium feeding to engulf diatom chains and protists several-fold larger than themselves, placing them in competition with copepods rather than with ciliates (Hansen and Calado, 1999; Jeong *et al.*, 2010). This nutritional plasticity confers a competitive edge under prey scarcity or nutrient limitation (Sherr and Sherr, 2007; Hansen, 2011).

The interaction and coexistence between ciliates and dinoflagellates are dynamic, shaped by a variety of environmental and biological factors. One intriguing ecological pattern observed across marine ecosystems is the seasonal duality or alternation in dominance between these two groups (Löder *et al.*, 2011; Gran-Stadniczeñko *et al.*, 2019; Jiang *et al.*, 2013; Armengol *et al.*, 2019). Early in a bloom, abundant pico- and nanoplankton fuel fast-dividing heterotrophic ciliates; later, as nutrients dwindle and the water column stratifies, mixotrophic or large heterotrophic dinoflagellates flourish—either photosynthesizing with stolen or permanent plastids or raptorially consuming senescent diatom chains—thereby completing the seasonal hand-off (Calbet et al., 2008; Hansen and Calado, 1999; Monti *et al.*, 2012).

Although this succession has been documented widely, considerable variability arises from geography, ecosystem type and local conditions. Understanding the drivers of these patterns is essential, not only ecologically and biogeochemically but also for anticipating how marine systems will respond to anthropogenic climate change.

Currently, marine ecosystems worldwide are experiencing profound shifts due to climate change. Long-term monitoring stations across latitudes reveal changes in the relative dominance of ciliates and dinoflagellates, with cascading effects on trophic interactions and biogeochemical cycling (Monti *et al.*, 2012; Verity and Borkman, 2010).

In this synthesis, I integrate evidence from temperate, polar and tropical seas to track seasonal shifts between ciliates and dinoflagellates, relate those shifts to phytoplankton-bloom stages and key physicochemical drivers, and place the emerging patterns in the context of long-term change under a warming, increasingly stratified ocean. To that end, I surveyed the literature—field observations, laboratory grazing and size-fractionation experiments, molecular profiles and environmental correlations—not to exhaustively meta-analyze the ~ 50 000 relevant papers, but to distill broad patterns that clarify the ciliate–dinoflagellate duality. Understanding these microbial community dynamics and their sensitivity to climate and environmental change is crucial, given the central role of microzooplankton in global carbon cycling, trophic interactions and ecosystem stability.

## OVERVIEW OF CILIATE AND DINOFLAGELLATE ECOLOGY

To better understand the ciliate-dinoflagellate duality, we first need to understand their basic differences and roles in the ocean.

### Trophic modes and roles

Among microzooplankton, ciliates and dinoflagellates usually account for most of the biomass and grazing, placing them at the center of pelagic food webs (Pierce and Turner, 1992; Sherr and Sherr, 2009). Despite frequent overlap in size, they rely on contrasting trophic tactics that partition resources and generate complementary—but sometimes competing—impacts on the plankton community.

**Fig. 2:**
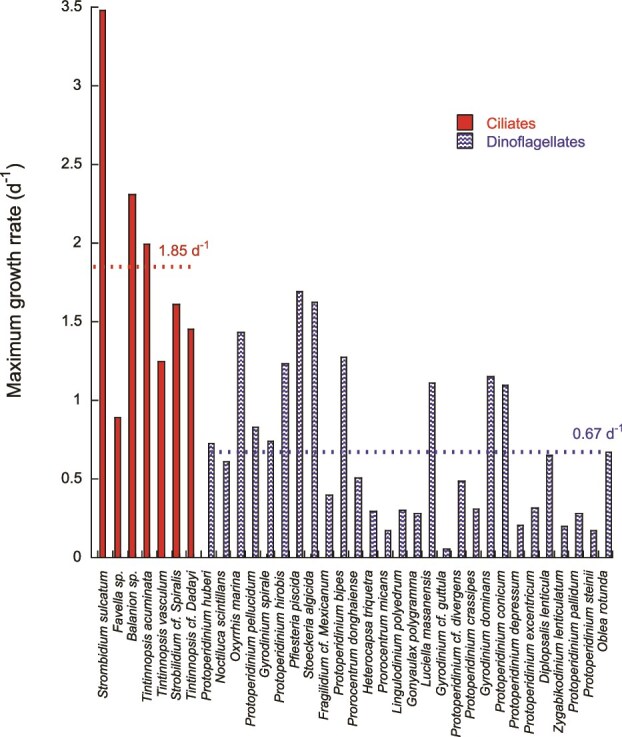
Maximum growth rates of different species of ciliates and dinoflagellates. Data from Sailley and Buitenhuis, 2014. The dotted line indicates the mean value for each group.

Ciliates sit toward the heterotrophic end of the spectrum. Most oligotrichs and tintinnids filter 2–12 μm particles (up to ~ 40 μm for tintinnids; Hansen *et al.*, 1994) with ciliary currents and can divide more than once per day when food is plentiful (Fig. 2). Such high intrinsic rates let them explode numerically during pico- or nanoplankton blooms, quickly curbing bacterial and minute algal populations (Fenchel, 1987; Vincent and Hartmann, 2001). Meta-analyses show that specific growth rate declines with cell volume in all protists, but ciliates keep a higher intercept: at ~ 10^3^ μm^3^ they divide roughly three times faster than heterotrophic dinoflagellates and still outpace them two- to three-fold above 10^5^ μm^3^ (Banse, 1982; Hansen *et al.*, 1997). A few genera (e.g. *Mesodinium*, *Strombidium*, *Laboea*, *Tontonnia*, *Pseudotontonia*) blur the line between trophic modes by retaining cryptophyte plastids, yet must keep grazing to replace fading kleptoplasts (Johnson *et al.*, 2007; Stoecker *et al.*, 2009; Mitra *et al.*, 2023).

Dinoflagellates span the full trophic continuum. Obligate photoautotrophs, versatile mixotrophs and raptorial heterotrophs coexist within the group, the latter able to engulf prey several times their own volume using pallium or peduncle feeding (Taylor, 1987; Hansen and Calado, 1999; Jeong *et al.*, 2010). Although their maximum division rates are generally lower than those of ciliates (Fig. 2), nutritional flexibility lets mixotrophic forms thrive when light is ample but dissolved nutrients or small prey are scarce, and it allows heterotrophs to boom when large, nutrient-rich diatoms appear (Stoecker, 1999; Burkholder *et al.*, 2008; Hansen, 2011). Maintaining chloroplasts, however, exacts a metabolic cost, so mixotrophs grow more slowly in pure heterotrophic mode than congeners lacking plastids (Jeong *et al.*, 2010).

Size-dependent prey choice sharpens their niche separation. Small heterotrophic dinoflagellates (<10^3^–10^4^ μm^3^) overlap the nanoplankton prey window exploited by ciliates and therefore compete directly with them. Larger raptorial dinoflagellates focus on diatom chains or protists beyond ciliate handling capacity, creating overlap with copepods instead (Hansen and Calado, 1999; Jeong *et al.*, 2010).

Through these complementary strategies, ciliates and dinoflagellates impose strong top–down control on microbial producers, regenerate large quantities of ammonium and other nutrients (Azam *et al.*, 1983; Caron, 2016), and mediate the efficiency and pathway of carbon flow toward mesozooplankton and fish larvae (Irigoien *et al.*, 2005; Calbet and Saiz, 2013). When the balance tilts—toward fast-growing ciliates after a surge of tiny prey or toward flexible dinoflagellates during and after diatom blooms—the architecture of the pelagic food web and its capacity for carbon export are reshaped accordingly.

### Morphological differences

Despite sharing dimensions, ciliates and dinoflagellates differ in their external structures, modes of locomotion and prey capture mechanisms (Fenchel, 1987; Montagnes *et al.*, 2008a). Marine pelagic ciliates are broadly categorized into aloricate (naked) and loricate (tintinnid) forms. Aloricate ciliates (e.g. oligotrichs, choreotrichs) are characterized by a flexible cell covering and abundant cilia that facilitate both swimming and feeding currents. They typically thrive in productive waters with high densities of small phytoplankton, though some species also persist in stratified or low-nutrient conditions (Dolan and Marrasé, 1995; Montagnes *et al.*, 2008b, Armengol *et al.*, 2019). Loricate ciliates, which can be abundant in neritic or coastal zones, are enclosed in a proteinaceous or composite lorica. Tintinnid species often exhibit prey selectivity and play a key role in structuring phytoplankton size spectra (Dolan and Marrasé, 1995; Dolan *et al.*, 2012). Most marine ciliates feed by filtering particles from the surrounding water using ciliary currents, which favors prey smaller than about one-tenth of the ciliate’s size (Fenchel, 1980, 1989; Jakobsen and Hansen, 1997).

Dinoflagellates traditionally fall into two main categories: athecate (naked) and thecate (armored). Athecate dinoflagellates, lacking rigid cellulose plates, are more agile and better suited to turbulent or variable waters but suffer greater predation risk. (Taylor, 1987; Martínez *et al.*, 2018; Jakobsen *et al.*, 2006). Thecate forms carry protective thecal plates that restrict motility; they therefore flourish in calm, stratified layers where their delicate pallium- or peduncle-feeding structures work best (Hansen and Calado, 1999; García-Oliva and Wirtz, 2022).

### Toxin production

Ciliates and dinoflagellates differ markedly in their ability to produce toxins, with profound ecological consequences for their roles in marine ecosystems. Dinoflagellates are well known for their capacity to produce a wide array of potent biotoxins, including saxitoxins, brevetoxins and okadaic acid, which are associated with harmful algal blooms (HABs) such as paralytic and diarrhetic shellfish poisoning events (Anderson *et al.*, 2012; Glibert, 2020). These toxins serve multiple ecological functions: at times they may deter grazers, inhibit the growth of competing phytoplankton, and can even affect microbial communities in their vicinity (Calbet *et al.*, 2002; Ma *et al.*, 2017; Mudge *et al.*, 2025). The ability to synthesize such compounds provides dinoflagellates with a competitive edge in environments where zooplankton grazing is otherwise a major limiting factor. For instance, toxin-producing genera like *Alexandrium* or *Karenia* can form persistent blooms by suppressing grazing by copepods and microzooplankton (Turner and Tester, 1997; Calbet *et al.*, 2003). In contrast, toxin production in ciliates is rare or poorly documented. While some soil ciliates may bioaccumulate toxins through their diet (Díaz *et al.*, 2006), there is no evidence that marine species may biosynthesize toxins. Instead, ciliates rely on rapid reproduction, behavioral escape responses and variable feeding strategies to evade predation and exploit ephemeral food resources (Jakobsen, 2001; Sailley and Buitenhuis, 2014). This fundamental difference means that ciliates may be more vulnerable to selective grazing, particularly by copepods or larger microzooplankton (Broglio *et al.*, 2004; Calbet and Saiz, 2005).

### Role as nutrient recyclers

Ciliates and dinoflagellates not only serve as critical trophic links in the marine food web but also play a major role in nutrient regeneration. By feeding on small prey, they assimilate organic matter and subsequently excrete or release nitrogen (N), phosphorus (P) and other essential elements in bioavailable forms (Sherr and Sherr, 1988; Bode *et al.*, 2004). This recycled pool of dissolved nutrients can be taken up again by primary producers, effectively “closing the loop” and supporting ongoing productivity in surface waters.

Ciliates can rapidly respond to increases in microbial prey and thereby enhance local recycling of nutrients through excretion (Caron and Hutchins, 2012). Meanwhile, many heterotrophic and mixotrophic dinoflagellates similarly release regenerated nutrients when they metabolize ingested cells, although some mixotrophs can also fix inorganic nutrients directly through photosynthesis (Stoecker *et al.*, 2009). Together, these activities help maintain a dynamic and efficient microbial loop, especially in oligotrophic or seasonally stratified conditions where external nutrient inputs are limited. For instance, in the NW Mediterranean, when present, ciliates accounted for the major portion of N regeneration (Selmer *et al.*, 1993). Even in upwelling ecosystems, such as that of A Coruña (NW Spain), microzooplankton contributes 33–43% to total N regeneration, whereas mesozooplankton contributes < 10% (Bode *et al.*, 2004). Thus, fluctuations in the relative abundances of ciliates and dinoflagellates can modulate nutrient cycling within coastal and open-ocean ecosystems, influencing both microbial community structure and overall productivity.

### Vertical distribution

Throughout the world’s oceans, the vertical positioning of planktonic ciliates and dinoflagellates is sculpted by a blend of behavioral traits—swimming speed, feeding mode, diel migration—and physical constraints such as light, nutrients, density gradients and shear. The outcome is a set of depth-specific communities that recur from oligotrophic gyres to productive estuaries (Dolan and Marrasé, 1995; Bhaskar *et al.*, 2020).

Small, agile aloricate ciliate forms (*Strombidium*, *Strobilidium*, *Balanion*) are typically concentrated in the euphotic zone where picoplankton and bacteria are abundant, whereas loricate tintinnids peak tens of meters deeper, often near the nutricline (Pitta and Giannakourou, 2000; Bhaskar *et al.*, 2020). Long vertical profiles from the Western Pacific to the abyssopelagic Arctic reveal similar stratification, with diversity and community composition shifting markedly from surface to deep-sea layers (Zhao *et al.*, 2017).

Many phototrophic dinoflagellate species undertake diel vertical migration: they ascend at night to nutrient-rich, well-lit surface layers for photosynthesis and descend by day to deeper strata to access additional nutrients or avoid predators (Cullen, 1985; Ji and Franks, 2007; Zheng *et al.*, 2023). The details are species- and water-column–dependent; in stratified laboratory columns, for example, *Heterocapsa triquetra* and *Prorocentrum minimum* adopt contrasting migration rhythms as salinity or temperature shifts (Jephson *et al.* 2011).

The superposition of ciliate depth sorting and dinoflagellate diel vertical migration often establishes a subsurface chlorophyll maximum or “thin layer” enriched in dinoflagellates and cryptophytes (Yentsch, 1965). Physical processes such as Langmuir circulation can subsequently aggregate or disperse these layers, seeding or suppressing harmful algal blooms (Kelly *et al.*, 2024). At finer scales, predator–prey coupling sharpens the vertical mosaic: in estuaries, ciliates such as *Favella* or *Balanion* track patchy dinoflagellate prey both horizontally and vertically, enhancing grazing efficiency and forming resource-rich zones exploited by larger zooplankton (Stoecker *et al.*, 1984).

Together, these observations show that vertical structure in the microzooplankton hinges on both intrinsic behavior—swimming, migration, prey tracking—and extrinsic forcing, from stratification to turbulence, with clear implications for food-web linkages and bloom dynamics.

## THE CENTRAL ROLE OF MIXOTROPHY

Mixotrophy is now recognized as common across most flagellated phytoplankton, so its mere presence offers dinoflagellates no automatic edge over other algae (Stoecker, 1999; Jeong *et al.*, 2010; Hansen, 2011). What distinguishes dinoflagellates and ciliates is the *form* that mixotrophy takes. Many dinoflagellates are constitutive mixotrophs that house their own plastids and photosynthesize continuously while grazing for nitrogen, phosphorus or micronutrients (Hansen, 2011). Others, such as *Dinophysis*, practice non-constitutive mixotrophy, sequestering foreign plastids or entire prey cytoplasms for days to weeks; this flexibility lets them flourish in high-light, low-nutrient layers and helps explain their prominence in harmful algal blooms (Mitra and Flynn, 2010; Anderson *et al.*, 2012). Ciliate mixotrophs, by contrast, rely almost exclusively on kleptoplasty: oligotrichs and tintinnids such as *M. rubrum*, *Laboea* and *Strombidium* steal cryptophyte chloroplasts and must continually replenish them, so the photosynthetic boost is modest and short-lived (Gustafson *et al.*, 2000; Johnson, 2011; Kim *et al.*, 2017).

Ecologically, mixotrophy blurs the sharp seasonal hand-off between ciliates and dinoflagellates, allowing both to linger into conditions that would otherwise exclude strict heterotrophs or autotrophs. It also short-circuits nutrient cycling: mixotrophic dinoflagellates divert prey-derived nitrogen and phosphorus directly into new photosynthetic biomass, whereas kleptoplastic ciliates achieve the same only briefly (Caron, 2016). Looking ahead, forecasts of stronger, longer stratification punctuated by episodic nutrient pulses seem poised to strengthen dinoflagellates with flexible, persistent mixotrophy, whereas ciliates may retain an edge chiefly in turbulent pockets or cryptophyte-rich refuges. Thus, it is the *mode* and *durability* of mixotrophy—rather than its mere presence—that shapes the ecological duality between ciliates and dinoflagellates.

## LATITUDINAL PERSPECTIVE

### Polar ecosystems

In polar and subpolar waters, extreme fluctuations in light and ice coverage overshadow temperature as the principal driver of biological production. Extended darkness and heavy ice cover in winter strongly constrain photosynthesis, forcing many microbes either to form resting cysts or to survive on minimal resources until the return of light. When the ice recedes in late winter or early spring, rapid stratification can fuel immense phytoplankton blooms, often dominated by diatoms or *Phaeocystis* spp., providing abundant prey for ciliate populations. Small aloricate forms (at times mixotrophic) quickly exploit surging nanoplankton and bacterial densities (Stoecker and Lavrentyev, 2018; Bhaskar *et al.*, 2020). For instance, in the Canadian Arctic, Onda *et al.* (2017) used 18S rRNA sequencing and microscopy data across seven years to show that surface communities during summer were dominated by smaller ciliates, particularly *Laboea strobila*, *Monodinium spp.* and *Strombidium spp.*, in association with low salinity and higher pico- and nanophytoplankton concentrations. In contrast, mixotrophic dinoflagellates were more prevalent at the subsurface chlorophyll maximum, where nitrate concentrations were higher. The authors also noted a shift after the 2007 sea ice minimum toward increased dominance of these small ciliates during the summer season. In Disko Bay, West Greenland, Levinsen *et al.* (2000) reported a pronounced seasonal succession pattern based on microscopy observations. The ciliate *Lohmanniela oviformis* was the first to respond to the phytoplankton peak in May, followed by large aloricate ciliates such as *Strobilidium spp.* and *Strombidium spp.* that peaked later, during the spring diatom bloom (April–May), simultaneously with dinoflagellates, primarily *Gyrodinium spirale* and *Protoperidinium spp*.

While several studies discuss the presence and roles of ciliates and dinoflagellates during phytoplankton blooms in the Southern Ocean, detailed data on their specific successional patterns throughout the bloom phases are limited. One study that provides some insight is by Swalethorp *et al.* (2019), which examined microzooplankton distribution in the Amundsen Sea Polynya during a *Phaeocystis antarctica* bloom. They observed that dinoflagellates accounted for up to 59% of the microzooplankton community, with ciliates also being significant contributors. However, the study primarily focuses on community composition at specific time points rather than detailing a temporal succession throughout the bloom. The same authors also conducted a 15-day mesocosm experiment simulating a *Phaeocystis* bloom. They showed that *Gymnodinium spp.* increased significantly, reaching a biomass of ~ 100 μg C l^−1^, while tintinnids were the primary ciliate responders, achieving only up to 8 μg C l^−1^. Other ciliates like *Strobilidium spp.* and *Strombidium spp.* declined over time. The results suggest that dinoflagellates, particularly mixotrophic forms, may be more competitive during intense *Phaeocystis* blooms, while ciliates may play a secondary role depending on bloom stage and prey availability.

Another relevant study shows that during late winter, the microzooplankton community within sea ice of the Weddell Sea (Antarctica) was rich in aloricate ciliates and foraminifers, particularly concentrated in the bottom 10 cm of the ice, with minimal presence in underlying seawater (Monti-Birkenmeier *et al.*, 2017). High biomass in the ice was correlated with elevated particulate organic carbon, nitrogen and salinity, while tintinnids, though present, were far less abundant than aloricate ciliates, indicating a tight link between microzooplankton structure and the winter microenvironment. However, in late summer, the microzooplankton community across Deception Island, Elephant Island and Antarctic Sound was dominated by tintinnids, especially in Elephant Island and Antarctic Sound where they comprised up to 83% of the community (Monti-Birkenmeier *et al.*, 2021). Their abundance was associated with surface and chlorophyll maximum depths and varied regionally due to hydrography (e.g. mixing layers, temperature gradients), with the highest abundance linked to relatively warmer, more mixed layers and elevated surface chlorophyll, suggesting coupling with phytoplankton structure and regional water mass properties.

Despite the presence or absence of general patterns, in many polar regions, there is marked interannual variability and sea ice often re-forms and light availability drops relatively early, causing both ciliates and dinoflagellates to subside quickly (Arrigo and Dijken, 2004). These compressed growing seasons highlight how sea-ice melt and light extremes are pivotal, with short-lived but intense ciliate proliferation fueled by diatom or *Phaeocystis* blooms, followed by an opportunity for dinoflagellates to take hold if physical stability persists long enough. In such polar waters, temperature rarely oscillates to the degree seen in temperate systems, so it is the interplay of ice dynamics, irradiance and nutrient depletion that sets the stage for ciliate–dinoflagellate alternation. At the same time, top–down pressures from copepods like *Calanus* spp. can rapidly limit ciliate biomass after the peak bloom period, often resulting in an apparent shift to dinoflagellates that are either less susceptible to predation or better equipped for low-nutrient conditions (Seuthe *et al.*, 2011).

### Temperate seas

A classic feature of temperate systems is the late winter-early spring phytoplankton bloom. This bloom usually drives a shift from ciliate-dominated microzooplankton communities in the cooler, turbulent months of late winter and early spring to dinoflagellate-dominated assemblages during the warmer, stratified conditions of late spring and summer (Margalef *et al.*, 1979; Calbet *et al.*, 2008; Glibert and Burford, 2017).

This seasonal progression has been documented in diverse temperate environments. The ecological rationale behind this pattern lies in the distinct physiological and trophic strategies of ciliates and dinoflagellates. Ciliates, particularly oligotrichs (e.g. *Strombidium*, *Strobilidium*) and tintinnids, are generally more successful in cooler (8–15°C), mixed waters where prey such as small flagellates and cryptophytes proliferate (Pierce and Turner, 1992; Montagnes *et al.*, 2008; Kamiyama, 2011). Their capacity for rapid population doubling, often within one to two days under optimal feeding conditions, may give them a competitive advantage during the beginning of the spring phytoplankton blooms (Banse, 1982; Strom and Morello, 1998; Ferreira *et al.*, 2022). Consequently, they frequently reach biomass maxima from March to April, coinciding with or shortly following the spring diatom bloom, as observed in many temperate seas (Fileman and Leakey, 2005; Aberle *et al.*, 2007).

These ciliate peaks are often short-lived but intense. For instance, Dolan and Marrasé (1995) observed marked ciliate biomass increases off the Catalan coast in early spring, corresponding to periods of well-mixed water and moderate temperatures around 13°C. Similarly, in Helgoland Roads, long-term monitoring reveals that mixotrophic ciliates respond rapidly to spring phytoplankton increases, thriving under the brief but favorable mixing and nutrient conditions typical of early spring (Yang *et al.*, 2014). Such dynamics are also seen in transitional zones or subtropical regions that experience spring-like nutrient inputs under relatively cool conditions, supporting transient ciliate dominance (Matsumoto *et al.*, 2021).

By the end of the bloom, particularly in those composed by long-chain diatoms, dinoflagellates may take over and deploy their array of trophic strategies to feed on the predominant large cells (Hansen and Calado, 1999; Calbet, 2008; Jeong *et al.*, 2021). As the season progresses, surface waters warm, vertical mixing diminishes and thermal stratification becomes established. This transition reduces the upward flux of nutrients from deeper layers, favoring smaller, slow-growing phytoplankton and altering the base of the food web (Margalef *et al.*, 1979; Smayda and Reynolds, 2001; Siokou-Frangou *et al.*, 2010). In such environments, mixotrophic dinoflagellates gain prominence due to their motility and ability to exploit nutrient gradients (Margalef, 1978; Hansen, 2011; Jeong *et al.*, 2021). Heterotrophic and mixotrophic genera such as *Gymnodinium*, *Gyrodinium*, *Protoperidinium*, *Dinophysis* and *Karlodinium* often dominate these warmer, stratified conditions by supplementing photosynthesis with ingestion of small algae and bacteria (Vila *et al.*, 2001; Calbet *et al.*, 2008; Jeong *et al.*, 2021). It is also during summer when dinoflagellates frequently form dense blooms. These blooms are facilitated by the motility and mixotrophic capabilities of many dinoflagellate species (and, at times, some mixotrophic ciliates as well), which allow them to exploit patchy nutrient distributions or ingest prey when inorganic nutrients are scarce (Hansen, 2011; Jeong *et al.*, 2010). Classic examples include blooms of *Alexandrium* spp. in the Gulf of Maine and the North Sea, often associated with paralytic shellfish poisoning events (Anderson *et al.*, 2005), *Alexandrium catenella and Gyrodinium impudicum* in the NW Mediterranean during summer (Vila *et al.*, 2001), and *Karenia* spp. in temperate coastal waters around the World (Brand *et al.*, 2012). These blooms are not only ecologically significant, often restructuring food webs and nutrient cycling, but can also pose risks to fisheries and human health through HAB events, especially when toxin-producing species dominate under prolonged stratified conditions.

Nevertheless, while this ciliate-to-dinoflagellate shift is a consistent trend across temperate seas, it is subject to substantial regional and interannual variability. Local physical and climatic events—such as storm-induced mixing, upwelling, monsoons or riverine inflows—can inject pulses of nutrients at atypical times, fostering diatom or cryptophyte blooms that provide favorable conditions for ciliates even during the stratified season (Monti *et al.*, 2012; Dhib *et al.*, 2013; Rekik *et al.*, 2023). For example, episodic nutrient inputs in the NW Mediterranean have been linked to unseasonal ciliate biomass peaks, driven by small microalgae proliferation (Rekik *et al.*, 2023).

Biological interactions further modulate the outcome of seasonal succession. Predation by mesozooplankton, such as copepods or even intraguild predation can differentially impact ciliates and dinoflagellates, either suppressing or facilitating their growth depending on timing, abundance and prey availability (Broglio *et al.*, 2004; Saiz *et al.*, 2007; Saiz *et al.*, 2013). High copepod grazing during phytoplankton blooms may reduce ciliate abundance, but as copepods switch to larger prey or migrate, small dinoflagellates may expand into the niche vacated by ciliates.

### Tropical ecosystems

By contrast, tropical and subtropical waters often feature permanent stratification, which keeps nutrients sequestered beneath a shallow mixed layer, leading to comparatively oligotrophic surface conditions (Karl, 1999). Primary production here is predominantly sustained by picophytoplankton—e.g. *Prochlorococcus* and *Synechococcus*—that support a fairly constant microzooplankton community (Olson and Daly, 2013; Schmoker *et al.*, 2014; Armengol *et al.*, 2019). Because temperature and mixing exhibit weaker seasonal variation, the classical “winter–spring–summer” pattern of ciliate–dinoflagellate successions observed in temperate regions is far less evident. Instead, a relatively stable background assemblage of both ciliates and dinoflagellates persists year-round, with mixotrophy proving especially advantageous for dinoflagellates in resource-poor waters (Stoecker, 1999; Hansen, 2011; Jeong *et al*., 2010). Although absolute densities can be lower than in nutrient-rich coastal zones, sporadic nutrient injections—caused by eddies, internal waves or tropical storms—may produce transient spikes in abundance (Olson and Daly, 2013). For instance, in a study of the tropical and subtropical Atlantic, Armengol *et al.* (2019) found that temperature and stratification strongly influenced the composition of the microzooplankton community. Dinoflagellates dominated the warm, stratified and nutrient-poor waters of the subtropical gyres, where deep thermoclines and stable conditions prevailed. In contrast, ciliates were more abundant in the cooler, nutrient-rich and less stratified upwelling regions off Cape Blanc and the Canary Current. This pattern reflects the ecological strategies of the two groups: dinoflagellates thrive in oligotrophic environments due to their mixotrophy and mobility, while ciliates benefit from the higher nutrient availability and prey densities typical of productive upwelling zones. However, a prominent role of mixotrophic ciliates, particularly in Polar waters, has also been suggested (Stoeker and Lavrentyev, 2018).

### Monsoon-related and upwelling areas

In monsoonal and upwelling-dominated systems (e.g. parts of the Arabian Sea, Bay of Bengal or eastern boundary currents), cyclic or episodic nutrient enrichment can blur typical “seasonal” categories (Garrison *et al.*, 2000). During strong wind forcing, coastal upwelling delivers nutrient-rich subsurface waters, triggering diatom blooms that favor a rapid buildup of microzooplankton consumers, often dominated by ciliates in the previous and early phases of both monsoon and bloom and by dinoflagellates at its peak (Stelfox *et al.*, 1999; Devi *et al.*, 2010; Zhong *et al.*, 2019; Elangovan and Gauns, 2021). Because such regions can experience multiple pulses of upwelling or monsoon-driven mixing within a single year, this succession may appear repeatedly on shorter timescales.

Taken as a whole, these latitudinal comparisons underscore the importance of recognizing how each marine system’s defining physical drivers—whether it be the presence and seasonal retreat of sea ice in polar latitudes, the nearly permanent stratification in tropical oligotrophic gyres, or the frequent pulsing of nutrients via upwelling or monsoonal winds—sculpt the resource landscapes for ciliates and dinoflagellates. Appreciating these cross-latitude patterns is critical for anticipating how climate change, sea-ice loss, altered wind systems or anthropogenic nutrient loading may shift the relative roles of these microzooplankton groups.

## MAJOR DRIVERS OF THE CILIATE-DINOFLAGELLATE DICHOTOMY

Although ciliates and dinoflagellates share overlapping ecological niches (both are protistan grazers on phytoplankton), they differ markedly in their thermal tolerances, feeding strategies and responses to changes in water column structure, nutrient flux and chemical parameters. Consequently, understanding how food availability, temperature, stratification, nutrient dynamics, salinity, pH, oxygen levels and biological interactions jointly influence ciliate and dinoflagellate communities is pivotal for predicting their seasonal and regional patterns.

### Nutrient availability

A primary environmental driver affecting food availability and composition is nutrient flux. When new nutrients (e.g. nitrate, phosphate, silicate) enter the euphotic zone—via mixing, upwelling or runoff—diatoms and other relatively large phytoplankton often proliferate. This pulse of big prey can favor heterotrophic dinoflagellates such as *Protoperidinium*, *Noctiluca* and certain *Gyrodinium* species that efficiently ingest chain-forming diatoms (Saito *et al.*, 2006; Sherr and Sherr, 2007). However, if nutrient pulses instead stimulate smaller species such as nanoflagellates, cryptophytes or other pico-sized cells, then ciliate populations (e.g. *Strombidium*, *Strobilidium* and many tintinnids) may expand more rapidly (Calbet *et al.*, 2008). These contrasts highlight how the same physical process—nutrient enrichment—can lead to divergent microzooplankton outcomes depending on the phytoplankton size spectrum that emerges.

Once nutrient inputs diminish and stratification increases, the phytoplankton community typically shifts toward smaller, sometimes more motile species that thrive in nutrient-depleted surface layers (Calbet *et al.*, 2008; Armengol *et al.*, 2019; Albin *et al.*, 2022). This reconfiguration of prey composition has two major impacts. First, it sustains a high turnover of small algae that ciliate grazers can exploit. Second, it sets the stage for mixotrophic dinoflagellates (e.g. *Karlodinium*, *Dinophysis*, *Gymnodinium*) to persist or even bloom because they can feed on nanoplankton or small ciliates while simultaneously performing photosynthesis under favorable light conditions (Jeong *et al.*, 2010; Stoecker *et al.*, 2017; Jeong *et al.*, 2021).

### Temperature

Comparative studies support the higher maximum growth rates of ciliates compared to heterotrophic dinoflagellates across various taxa and cell sizes, indicating an inherent physiological advantage at elevated temperatures (Strom and Morello, 1998; Ferreira *et al.*, 2022). This seems to contradict their distribution patterns in colder waters (Franze and Lavrentyev, 2014; Armengol *et al.*, 2019). However, the degree of response to temperature (i.e. Q_10_) is not the only factor explaining the distribution of a species. The key here is to observe their thermal optimum and sensitivity. In this respect, recent research shows marine ciliates typically exhibit lower thermal sensitivity compared to freshwater ciliates, reflecting adaptations to more thermally stable marine environments (Lukić *et al*., 2022).

Dinoflagellates, conversely, display more variable responses to temperature, closely linked to their trophic strategy. Mixotrophic dinoflagellates, such as *Biecheleria cincta* and *Yihiella yeosuensis*, show optimal growth within moderate temperature windows (15–25°C), with peak growth rates occurring around 25°C (Kang *et al.*, 2020; You *et al.*, 2023). The toxin-producing *Gymnodinium catenatum* similarly exhibits optimal growth between 20°C and 29°C, with cell division ceasing at temperatures below 15°C (Aboualaalaa *et al.*, 2024). In contrast, some dinoflagellates, particularly those preying on large diatoms or possessing mixotrophic capabilities, exhibit broader thermal tolerances and may even thrive in relatively cooler waters if adequate prey availability persists (Jeong *et al.*, 2010; You *et al.*, 2023).

Temperature impacts extend beyond growth to the ecological roles of ciliates and dinoflagellates. Moderately elevated temperatures have been linked to increased carbon fixation rates and altered cellular elemental ratios (C:N) of dinoflagellates, indicating significant metabolic adjustments that could influence marine biogeochemical cycles under climate warming scenarios (Wen *et al.*, 2025). Additionally, harmful algal bloom species such as *Ostreopsis ovata* demonstrate accelerated biomass accumulation at elevated temperatures (26–30°C), although their toxin production can peak at moderately lower temperatures (20–22°C), highlighting the complex relationship between temperature, growth and toxicity (Granéli *et al.*, 2011).

**Fig. 3:**
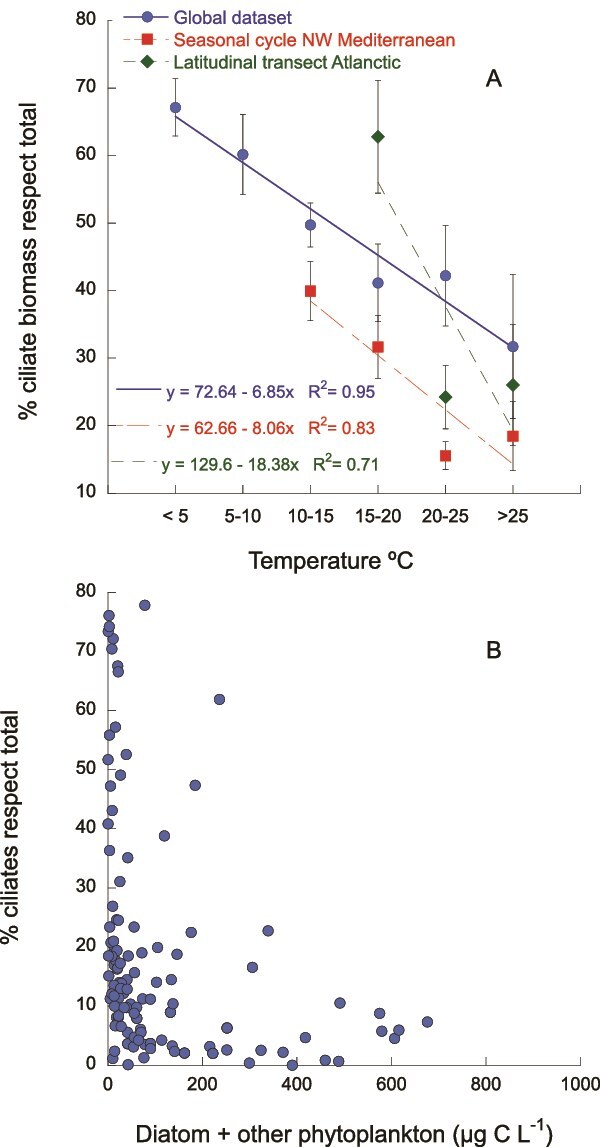
Relationship between temperature (A) and the percentage of ciliates respect the total microplankton community (ciliates + dinoflagellates) in a global dataset (Saiz and Calbet, 2007), a seasonal study (PUDEM long-term series) in the NW Mediterranean off Barcelona, Spain (Arin et al., unpublished) and a latitudinal transect across the Atlantic (Armengol *et al.,* 2019). Relationship total prey abundance (B) and the percentage of ciliates with respect to the total microplankton community in a global dataset (Saiz and Calbet, 2007).

Some field observations support these laboratory findings, showing that, generally—whether across seasonal cycles, latitudinal transects or in the global ocean—ciliates tend to dominate at lower temperatures, while dinoflagellates show the opposite trend (Fig. 3a). However, temperature rarely acts as the sole determining factor; instead, it interacts with other variables such as nutrient availability, prey composition and physical mixing. Therefore, it remains unclear whether temperature alone drives this pattern or if it results from a combination of interacting factors. Notably, no consistent relationship emerges when phytoplankton abundance is considered as a potential driver of ciliate or dinoflagellate dominance (Fig. 3b).

### Water stability

Moderate turbulence benefits many ciliates by increasing encounter rates with motile prey; for instance, small oligotrichs can exploit weak mixing to capture diverse nanoplankton efficiently (Shimeta *et al.*, 1995; Dolan *et al.*, 2003; Havskum, 2003; Martínez *et al.*, 2018). However, excessive turbulence can disrupt ciliate swimming and feeding, diminishing their net growth. By contrast, many dinoflagellates prefer calmer, stratified water columns because turbulence can interfere with their motility and formation of thin layers where prey aggregates (Smayda and Reynolds, 2001). Moreover, stable, low-turbulence environments facilitate vertical migration and mixotrophy in dinoflagellates, as they can move across nutritional gradients to supplement autotrophy with heterotrophy (Stoecker, 1999; Jeong *et al.*, 2010).

### Salinity

Dinoflagellates generally exhibit broad ecological tolerance to variations in salinity, enabling them to inhabit diverse marine environments from freshwater-influenced estuaries to highly saline oceanic waters. Their adaptability is facilitated by flexible physiological mechanisms, allowing survival and growth across wide salinity ranges. For instance, invasive dinoflagellates such as *P. minimum* readily adjust to lower salinities by increasing RNA synthesis and DNA replication, demonstrating significant ecological plasticity (Skarlato *et al.*, 2018). Similarly, the mixotrophic dinoflagellate *Paragymnodinium shiwhaense* maintains optimal growth at wide salinity ranges (20–40 PSU), indicative of broad tolerance and physiological flexibility (Jeong *et al.*, 2018). Furthermore, closely related heterotrophic dinoflagellates within the genus *Gyrodinium* (*Gyrodinium dominans*, *G. jinhaense* and *G. moestrupii*) show differential distribution patterns in response to salinity, suggesting subtle but important niche differentiation along salinity gradients (Lee *et al.*, 2021).

Beyond mere tolerance, specific physiological processes in dinoflagellates, such as bioluminescence and toxin production, also display clear responses to salinity. Bioluminescent species, including *Noctiluca scintillans*, *Polykrikos kofoidii* and *Alexandrium mediterraneum* increase their bioluminescent intensity significantly as salinity rises from estuarine (10 PSU) to oceanic levels (ca. 40 PSU), suggesting enhanced defensive responses in more saline waters (Park *et al.*, 2024). Toxin-producing dinoflagellates such as *Alexandrium* spp. demonstrate an optimal salinity range for toxin synthesis and growth, with reduced performance at both higher and lower salinity extremes, indicating that salinity is a critical environmental factor influencing toxin biosynthesis pathways (Bui *et al.,* 2024).

In contrast to dinoflagellates, ciliates seem to exhibit more clearly delineated and specialized responses to salinity gradients. Ciliate communities, particularly tintinnids, often segregate distinctly into freshwater, mid-estuarine and marine communities, suggesting narrower and more specific salinity preferences and niche separations (Albergoli and Alder, 2024). These community shifts are frequently accompanied by distinct physiological adaptations, as demonstrated in *Euplotes vannus*, which undergoes significant changes in growth dynamics and energy metabolism when subjected to acute versus chronic salinity stress (Li *et al.*, 2024). Thus, ciliates typically display a higher sensitivity and clearer community restructuring along salinity gradients, distinguishing their ecological response patterns from the broader, more flexible salinity tolerance exhibited by dinoflagellates.

### Predation

Biological interactions add a further layer of complexity. Selective copepod grazing can significantly influence the distribution and composition of microzooplankton communities, particularly ciliates and dinoflagellates. Field and experimental studies consistently show that copepods, including genera like *Paracalanus* and *Acartia*, often exhibit a clear feeding preference for ciliates over phytoplankton, but may even prefer heterotrophic dinoflagellates in certain contexts (Suzuki *et al.*, 1999; Broglio *et al.*, 2004; Calbet and Saiz, 2005; Sommer *et al.*, 2005). This selective predation is influenced by prey motility, size, and nutritional value and may lead to significant trophic impacts despite relatively low overall predation pressure (Broglio *et al.*, 2004). This preference can suppress certain ciliate taxa and promote phytoplankton groups that escape both micro- and mesozooplankton grazing, indirectly altering the competitive balance among microzooplankton and primary producers (Vincent and Hartmann, 2001). Simultaneously, some large dinoflagellates may prey on smaller ciliates, adding a predatory layer within the microzooplankton community (Jeong *et al.*, 2010 and references therein).

Selective grazing behaviors thus reshape planktonic food web structures, influencing seasonal succession patterns and potentially altering ecosystem trophic efficiency and biogeochemical cycling.

### Ocean acidification

Over broader temporal scales, ocean acidification (OA) is expected to exert divergent effects on planktonic protists. Calcifying protists are anticipated to be negatively impacted due to reduced carbonate saturation states and the energetic costs associated with maintaining calcified structures under low pH conditions (Balch and Utgoff, 2009; Xu *et al.*, 2023). Conversely, some mixotrophic dinoflagellates may exhibit a high degree of physiological plasticity, enabling them to maintain growth and photosynthetic activity under acidified conditions. For instance, *Prorocentrum donghaiense* increased its photochemical efficiency and antioxidant defenses under OA, thereby alleviating the adverse effects of nanoplastics (Zhu *et al.*, 2024). Similarly, rising pCO₂ levels have been shown to stimulate growth and toxicity in *Alexandrium minutum*, suggesting that OA may enhance the ecological success and risk of harmful algal blooms (Lian *et al.*, 2022). Other bloom-forming species, such as *Scrippsiella trochoidea* or *Alexandrium tamarense*, also demonstrate species-specific responses, with some maintaining growth across a wide pCO₂ range through adjustments in carbon acquisition mechanisms (Eberlein *et al.*, 2014). OA also impacts marine ciliates, although primarily through indirect mechanisms. Most studies have found that while ciliates are relatively tolerant to decreased pH levels, their ecological performance can be significantly affected by OA-induced changes in their phytoplankton prey. For instance, increased CO₂ levels can alter prey cell size, morphology and nutritional quality, which in turn modifies ciliate ingestion rates and feeding behavior (Kendall, 2015; Olson *et al.*, 2018). *Eutintinnus* sp., for example, showed enhanced ingestion rates when fed phytoplankton grown under elevated pCO₂ due to increased cell volume (Olson *et al.*, 2018). These changes may lead to shifts in ciliate feeding dynamics and community interactions.

Moreover, large-scale mesocosm experiments have demonstrated that while direct effects of OA on microzooplankton like ciliates are minimal, significant restructuring of plankton communities can occur through bottom-up effects mediated by altered primary producers (Aberle *et al.**,*** 2013; Horn *et al.*, 2016; Spisla *et al.*, 2021). Thus, although ciliates may not suffer direct physiological harm from acidification, the cascading effects of OA on planktonic food webs could profoundly influence their ecological roles.

In contrast, elevated pH levels—often observed during intense phytoplankton blooms in coastal waters—can impose significant constraints on both autotrophic and heterotrophic protists. Studies have shown that high pH (above 9) can directly impair cellular processes, including enzyme function and membrane stability, leading to reduced growth rates or even mortality in many species (Pedersen and Hansen, 2003a, 2003b; Hansen *et al.*, 2007). In natural plankton communities, elevated pH has been associated with decreased species richness and selective survival of a few tolerant taxa, which can persist even at pH levels above 9.5 (Pedersen and Hansen, 2003a). Experimental incubations also revealed that most heterotrophic protists, particularly ciliates and dinoflagellates, suffer significant mortality under prolonged high pH exposure (Pedersen and Hansen, 2003b). Furthermore, growth of common red-tide dinoflagellates like *H. triquetra* and *P. minimum* is constrained more by elevated pH itself than by the availability of inorganic carbon, indicating that direct pH effects can override DIC limitations in certain bloom scenarios (Hansen *et al.*, 2007).

Future climate scenarios combining warming (+3°C) and acidification (−0.4 pH units) significantly altered the timing and abundance of marine microzooplankton groups. In mesocosm experiments, ciliate and autotrophic dinoflagellate biomass peaked earlier but reached lower magnitudes under combined warming and acidification compared to nutrient-only treatments (Calbet *et al*., 2014). Ciliates were particularly sensitive to low pH, showing reduced abundance likely due to declines in prey quality rather than direct acidification stress. Warming further exacerbated this effect by reducing their growth efficiency, shifting more energy toward respiration and away from biomass accumulation. As a result, ciliate dominance was shortened and delayed relative to favorable nutrient-only conditions. Dinoflagellates, including *G. spirale*, also experienced earlier but lower peaks under combined stressors. While their broader temperature tolerance allowed for some resilience, the synergistic impact of acidification and warming limited their ecological success and altered typical bloom dynamics. Overall, the combination of warming and acidification not only advanced bloom timing but also reduced the persistence and biomass of key microzooplankton groups, disrupting their succession and diminishing trophic transfer efficiency (Calbet *et al.*, 2014).

## CONCLUDING REMARKS ON FUTURE ECOSYSTEMS

**Fig. 4:**
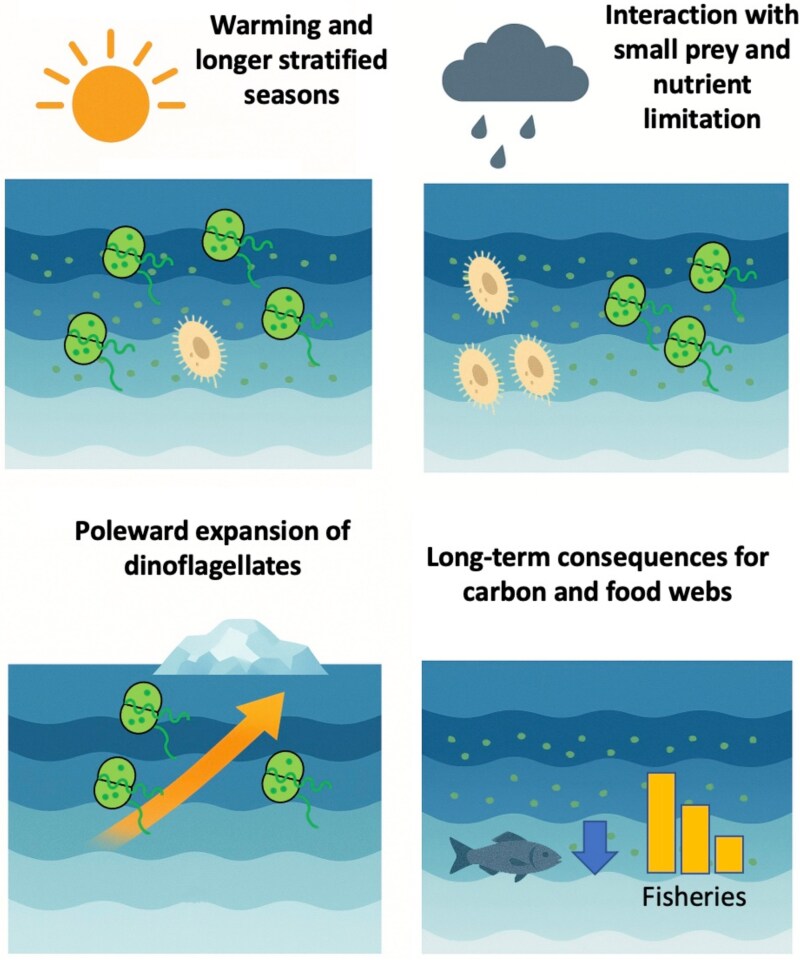
Schematic of the main climatic effects on the future ciliate and dinoflagellate populations.

Global climate change projections consistently point to higher sea-surface temperatures, stronger stratification and increased frequency of nutrient-poor conditions in many marine environments (Bopp *et al.*, 2013; IPCC, 2023). These alterations influence both trophic interactions and the balance between ciliates and dinoflagellates, especially in systems that are historically characterized by periodic mixing events or nutrient pulses. A key factor is that warming interacts with nutrient limitation to favor smaller phytoplankton (Finkel *et al.*, 2010), mixotrophy, and stable, low-turbulence conditions—all of which tend to impact the seasonal prominence of ciliates versus dinoflagellates in distinct ways (Fig. 4).

### Warming and longer stratified seasons

Warming is predicted to prolong stratification in temperate and tropical seas, as well as reduce sea-ice coverage in polar regions (Coma *et al.*, 2009; Kumar *et al.*, 2020). From a microzooplankton perspective, longer stratified periods often benefit motile, mixotrophic dinoflagellates. In contrast, strictly heterotrophic ciliates depend heavily on pulses of small phytoplankton to fuel rapid population growth. With a more prolonged stratified season, nutrient inputs from deeper layers diminish; thus, the small algae that otherwise sustain ciliate peaks can become scarcer or appear in short bursts. Over time, this scenario may foster recurrent dinoflagellate blooms or stable dinoflagellate populations, especially in oligotrophic waters (Caron and Hutchins, 2012; Barton *et al.*, 2016).

In Mediterranean regions, light availability, already high in summer, may interact with these extended stratified phases to bolster mixotrophic feeding in dinoflagellates. Mesocosm research in Eastern Mediterranean waters (Calbet *et al.*, 2012) has shown that high light availability can enhance mixotrophic strategies in oligotrophic settings, enabling dinoflagellates to thrive when nutrients are depleted in surface layers. Although bloom-forming ciliates, particularly those capable of kleptoplastidy (e.g. *M. rubrum*), can also benefit from strong sunlight, they often depend on specific cryptophyte prey to maintain chloroplast function (Esteban *et al.*, 2010; Johnson *et al.*, 2023). Consequently, a future characterized by warming and stable, high-light conditions could tip the scales toward mixotrophic dinoflagellates (and perhaps some generalist mixotrophic ciliate species) that do not rely on a single type of ingested chloroplast.

### Interaction with small prey and nutrient limitation

Multiple studies of plankton food webs under future ocean conditions suggest that a shift toward smaller average phytoplankton will magnify the role of microzooplankton grazers (Daufresne *et al.*, 2009; Onda *et al.*, 2017), particularly in productive ecosystems (Chen *et al.*, 2012). On one hand, ciliates excel at feeding on pico- and nano-sized algae, and their quick generation times could allow them to rapidly graze these prey whenever nutrients are briefly reintroduced. On the other hand, persistent oligotrophy, typical of a strongly stratified ocean, tends to favor mixotrophic dinoflagellates, which do not rely exclusively on high prey density (Stoecker, 1999; Calbet *et al.*, 2012). Hence, the net outcome for ciliates *versus* dinoflagellates partly hinges on how often storms or upwelling pulses briefly deliver nutrients and how strong those events are in a warming ocean.

### Poleward expansion of dinoflagellates

As temperatures rise, many warm-adapted dinoflagellates—including some harmful algal bloom (HAB) taxa—are predicted to shift poleward (Barton *et al.*, 2016; Borges *et al.*, 2022; Kléparski et al., 2024). In high-latitude seas, new dinoflagellate arrivals may exploit longer ice-free seasons and, once established, maintain populations in strongly stratified surface layers (Barton *et al.*, 2016; Borges *et al.*, 2022; Kléparski *et al.*, 2024). This could gradually displace or constrain ciliate-dominated systems during late summer, even in the sub-Arctic or northern temperate zones, further highlighting the interplay of warming, changing prey fields and species introductions.

### Long-term consequences for carbon and food webs

If climate warming leads to prolonged intervals of low turbulence and oligotrophy, we can expect a stronger microbial loop featuring dinoflagellates dominating in permanently stratified conditions (Caron and Hutchins, 2012). Some ciliates, particularly large loricate forms, may see more frequent top–down pressure if copepod populations align with earlier, smaller spring blooms. Meanwhile, dinoflagellates may form more frequent or more extended blooms, sometimes toxic, if not suppressed by grazing (Calbet *et al.*, 2003; Ladds and Gobler, 2025).

Moreover, small-scale turbulence studies (Shimeta *et al.*, 1995; Dolan *et al.*, 2003; Havskum, 2003; Martínez *et al.*, 2018) show that short-lived bursts of mixing can temporarily boost growth on selected species of microzooplankton, but negatively affect many others. However, in a future ocean with warming and sporadic extreme weather, these turbulence-driven gains may be short-lived and overshadowed by longer calm periods that favor dinoflagellates specialized for stable layers. Thus, while climate extremes might briefly promote high ciliate feeding rates, the broader trend of reduced net mixing and extended stratification still tips the balance toward dinoflagellates.

From a biogeochemical viewpoint, these changes in ciliate–dinoflagellate dynamics may have repercussions for carbon flux, nutrient remineralization and higher-trophic-level productivity. Over time, intensified microzooplankton grazing in a warm, stratified ocean shortens the residence time of phytoplankton biomass, while also promoting nutrient recycling near the surface (Taylor, 1982), which could have knock-on effects for fisheries productivity and benthic-pelagic coupling. Meanwhile, a consistent presence of dinoflagellates—including toxic or HAB-forming taxa—under stable, high-temperature conditions may pose additional threats to fisheries and aquaculture (Gobler, 2020; Wells *et al.*, 2020).

In summary, while global warming and stratification trends often benefit dinoflagellates overall, ciliates retain key advantages where short-lived nutrient pulses lead to booms of small algae or moderate turbulence helps sustain ciliate feeding. Indeed, long-term datasets (Verity and Borkman, 2010; Monti *et al.*, 2012) show that modern microzooplankton communities have undergone structural shifts, with ciliates, particularly aloricate forms, gaining prominence in some regions, whereas heterotrophic dinoflagellates have also become increasingly important, especially under changing nutrient regimes and extended warm, stratified periods. Given the broad taxonomic diversity and short generation times of both ciliates and dinoflagellates, adaptive capacity in these groups (Calbet and Saiz, 2022) can be high, buffering some ecosystem changes. Yet the precise relative success of ciliates *versus* dinoflagellates in a future ocean will be determined by the complex interplay of local hydrography, nutrient supply and biological interactions, including top–down predation by mesozooplankton or intraguild predation by large heterotrophic dinoflagellates (Broglio *et al.*, 2004; Jeong *et al.*, 2010; Calbet and Saiz, 2013).

The consequences of shifting ciliate-dinoflagellate balances extend beyond microplankton ecology. Changes in their relative dominance, trophic roles or vertical distribution—especially in response to warming, acidification or altered mixing regimes—could ripple through entire ecosystems, affecting not just microbial loop dynamics but also the timing and magnitude of primary production, zooplankton recruitment and pelagic-benthic coupling. Indeed, the efficiency of the biological carbon pump and the resilience of oceanic food webs may hinge on the fine-scale dynamics of these small but ecologically potent protists (Caron, 2016; Calbet and Saiz, 2013).

Looking ahead, the duality between ciliates and dinoflagellates should not be viewed as a rigid seasonal template but as a dynamic continuum shaped by interacting abiotic and biotic variables. Future research must move beyond correlative studies to mechanistically disentangle how trophic plasticity, environmental forcing and biological interactions merge to drive community shifts. Long-term time-series programs, combined with high-resolution molecular and experimental approaches, will be crucial in this effort, particularly as we strive to predict the cascading effects of climate change on planktonic systems. Ultimately, understanding the nuanced roles of ciliates and dinoflagellates in marine ecosystems remains not only a fundamental question in microbial ecology but also a key to anticipating the resilience and trajectory of the ocean under rapid environmental transformation.

## Data Availability

The dataset is available in the DIGITAL.CSIC repository: 10.20350/digitalCSIC/17521.
